# A Deep-Learning-Enhanced Ultrasonic Biosensing System for Artifact Suppression in Sow Pregnancy Diagnosis

**DOI:** 10.3390/bios16020075

**Published:** 2026-01-27

**Authors:** Xiaoying Wang, Jundong Wang, Ziming Gao, Xinjie Luo, Zitong Ding, Yiyang Chen, Zhe Zhang, Hao Yin, Yifan Zhang, Xuan Liang, Qiangqiang Ouyang

**Affiliations:** 1 Third Affiliated Hospital of Sun Yat-sen University, Guangzhou 510630, China; 2 Intelligent Vehicle College, Guangzhou Polytechnic University, Guangzhou 511483, China; 3 College of Electronic Engineering, South China Agricultural University, Guangzhou 510642, China

**Keywords:** ultrasonic biosensor, artificial intelligence, deep learning, YOLOv8, artifact removal, sow pregnancy diagnosis, ultrasound imaging

## Abstract

The integration of artificial intelligence (AI) with ultrasonic biosensing presents a transformative opportunity for enhancing diagnostic accuracy in agricultural and biomedical applications. This study develops a data-driven deep learning model to address the challenge of acoustic artifacts in B-mode ultrasound imaging, specifically for sow pregnancy diagnosis. We designed a biosensing system centered on a mechanical sector-scanning ultrasound probe (5.0 MHz) as the core biosensor for data acquisition. To overcome the limitations of traditional filtering methods, we introduced a lightweight Deep Neural Network (DNN) based on the YOLOv8 architecture, which was data-driven and trained on a purpose-built dataset of sow pregnancy ultrasound images featuring typical artifacts like reverberation and acoustic shadowing. The AI model functions as an intelligent detection layer that identifies and masks artifact regions while simultaneously detecting and annotating key anatomical features. This combined detection–masking approach enables artifact-aware visualization enhancement, where artifact regions are suppressed and diagnostic structures are highlighted for improved clinical interpretation. Experimental results demonstrate the superiority of our AI-enhanced approach, achieving a mean Intersection over Union (IOU) of 0.89, a Peak Signal-to-Noise Ratio (PSNR) of 34.2 dB, a Structural Similarity Index (SSIM) of 0.92, and clinically tested early gestation accuracy of 98.1%, significantly outperforming traditional methods (IoU: 0.65, PSNR: 28.5 dB, SSIM: 0.72, accuracy: 76.4). Crucially, the system maintains a single-image processing time of 22 ms, fulfilling the requirement for real-time clinical diagnosis. This research not only validates a robust AI-powered ultrasonic biosensing system for improving reproductive management in livestock but also establishes a reproducible, scalable framework for intelligent signal enhancement in broader biosensor applications.

## 1. Introduction

The strategic integration of advanced technology into agriculture is pivotal for enhancing productivity and sustainability, as underscored by national policies aiming to build a robust agricultural sector through modern science and equipment. In the swine industry, reproductive efficiency is a primary determinant of profitability and food security. Accurate and early pregnancy diagnosis in sows is therefore critical, allowing for timely management decisions that optimize farrowing rates and resource allocation [1,2,3].

Ultrasound imaging has emerged as a cornerstone technology for non-invasive pregnancy diagnosis in veterinary medicine [4,5]. As a biosensor, the ultrasound probe transmits high-frequency sound waves into tissue and receives the reflected echoes, converting these mechanical signals into electrical data that form an image. The core principle relies on the differential reflection of ultrasound at interfaces between tissues with distinct acoustic impedances [6,7]. In sow pregnancy detection, this enables the visualization of embryonic structures, such as gestational sacs and fetuses, within the uterus. However, the fidelity of these images is often compromised by various acoustic artifacts, including reverberation, acoustic shadowing, and side lobes [8,9]. These artifacts arise from the physical principles of ultrasound propagation—such as multiple reflections, attenuation, and scattering—and can obscure critical anatomical features, leading to diagnostic uncertainty, especially during early gestation [10].

Traditional digital signal processing techniques, including spatial filtering and Fourier-based methods, have been employed to mitigate artifacts but often face a fundamental trade-off between artifact suppression and the preservation of diagnostically crucial tissue boundaries and textural details [10]. Their reliance on manually engineered filters limits their adaptability to the complex and variable nature of acoustic artifacts in biological tissues [11].

The recent ascendancy of artificial intelligence (AI), particularly Deep Neural Networks (DNNs), offers a paradigm shift. DNNs possess an exceptional capacity for adaptive feature extraction and can learn complex, non-linear mappings from noisy inputs to clean outputs directly from data [12,13]. In medical imaging, models like YOLOv8 have demonstrated remarkable performance in real-time object detection and image enhancement tasks [14,15]. Their application to ultrasound biosensing can potentially disentangle genuine tissue signals from artifact-induced interference in a data-driven manner, moving beyond the limitations of pre-defined filter models.

This study bridges the gap between AI-driven image analysis and fundamental ultrasonic physics by developing an intelligent biosensing system. We posit that a deep learning model, trained on a physically representative dataset and integrated with a standard ultrasonic biosensor (mechanical sector-scanning B-mode ultrasound system), can significantly enhance image quality for sow pregnancy diagnosis. Our work makes the following key contributions: (1) Biosensor-Centric System Design: We detail the integration of a mechanical sector-scanning ultrasound probe as the primary biosensor with a DNN-based processing backend. (2) Data-driven AI Model: We develop and train a YOLOv8-based DNN model on a dataset encompassing common ultrasound artifacts, for the simultaneous tasks of artifact suppression and anatomical structure annotation in sow ultrasound images. (3) Comprehensive Performance Validation: We quantify the performance gains of our AI-enhanced system against traditional methods using standard metrics (IoU, PSNR, SSIM) and processing speed, demonstrating its clinical viability. (4) An Effective Framework for Ultrasound Enhancement: We establish a reproducible framework that combines ultrasonic physical principles with data-driven AI optimization, offering a blueprint for enhancing various biosensing applications.

## 2. Experimental Section: System and Principles

### 2.1. The Ultrasonic Biosensing System: An Overview

**Figure 1:**
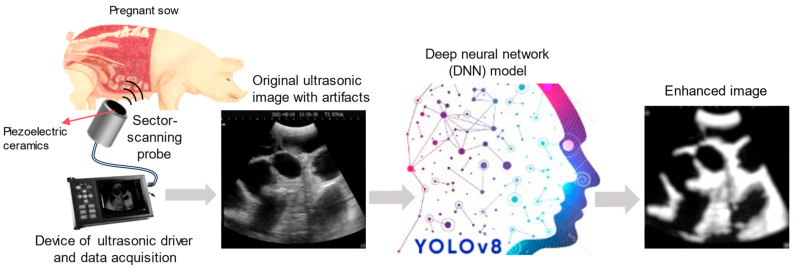
Schematic of the AI-enhanced ultrasonic biosensing system. The process begins with the ultrasound biosensor (probe) emitting pulses and receiving echoes from the sow’s abdomen. The raw radiofrequency (RF) data is beamformed into a B-mode image in the polar coordinate system. This image, often corrupted by artifacts, is then fed into the trained DNN model (YOLOv8). The AI model performs intelligent processing to suppress artifacts and annotate key regions of interest (e.g., gestational sac). The final output is a clean, enhanced image with diagnostic annotations, displayed for the user.

The core of our experimental setup is a biosensing chain that begins with a mechanical sector-scanning B-mode ultrasound system. This system acts as the data acquisition front end, whose raw output is then intelligently processed by our custom DNN model. The complete workflow, from signal generation to enhanced image output, is illustrated in Figure 1. The DNN model performs two complementary tasks: (1) detection and segmentation of artifact regions and anatomical structures; (2) generation of an enhanced visualization through artifact masking and structure annotation, providing clinicians with a cleaner, more interpretable diagnostic view.

### 2.2. Principles of the Ultrasonic Biosensor

The physical foundation of our biosensor rests on the pulse-echo principle [16,17]. The probe, containing a piezoelectric crystal, converts electrical pulses into mechanical ultrasound waves (typically 2–15 MHz for abdominal imaging). These waves propagate through tissues, and at interfaces with differing acoustic impedance Z (Equation (1)), a portion of the energy is reflected back to the probe.

(1) Z=ρc

where ρ is tissue density and c is the speed of sound (~1540 m/s in soft tissue). The reflection coefficient R for normal incidence is given by:

(2) $$R\;={\;(\frac{Z_{2}\;-\;Z_{1}}{Z_{2}\;+\;Z_{1}})}^{2}$$

The returning echoes are converted back into electrical signals. The depth d of a reflector is calculated from the time delay Δt between pulse emission and echo reception:

(3) $$d\;=\;\frac{c\cdot \Delta t}{2}$$

A sequence of these A-mode (amplitude vs. depth) lines is acquired by mechanically or electronically sweeping the ultrasound beam to form a two-dimensional B-mode (brightness-mode) image, where the amplitude of the echo is displayed as a pixel brightness.

### 2.3. Biosensor Hardware: Mechanical Sector-Scanning Probe

**Figure 2:**
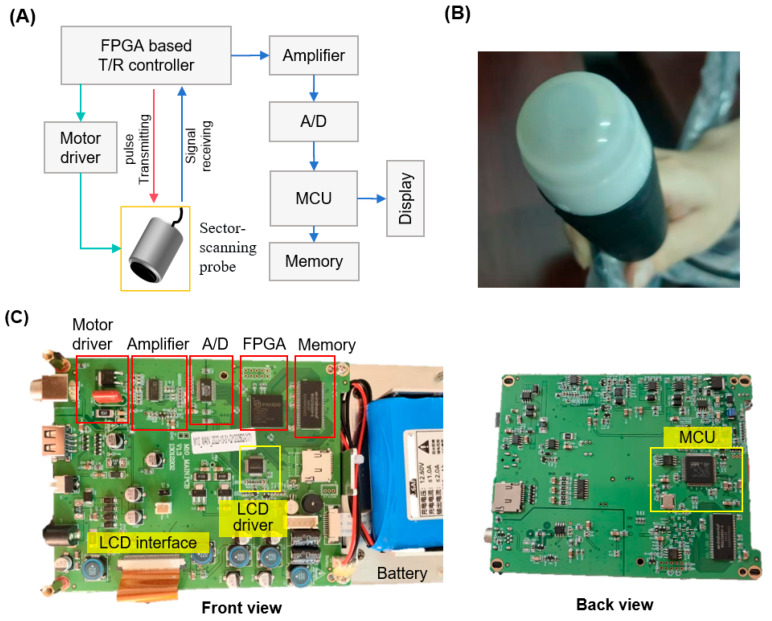
Mechanical sector-scanning biosensor assembly. (**A**) Diagram of the oscillating sector-scanning probe sensor system, showing the motor-driven Piezoelectric ceramics that sweeps through a sector. (**B**) Photograph of the actual mechanical sector-scanning probe used for data acquisition. (**C**) Photograph of the T/R control and data acquisition board of signal path, from pulse transmission on the FPGA-based circuit module to image display.

For this study, we employed a (oscillating mechanical sector-scanning B-ultrasound system). As shown in Figure 2, its probe uses a single piezoelectric crystal oscillated by a motor through a 30–90° arc. This mechanical sweeping generates a fan-shaped imaging sector, ideal for intercostal and abdominal imaging in animals like sows [18]. The system’s circuit board, built around a large-scale Field-Programmable Gate Array (FPGA), manages multi-channel ultrasound transmission, variable frequency control, and high-speed signal processing using low-voltage CMOS components. This design ensures low power consumption, minimal thermal drift, and high reliability—key attributes for a robust biosensing platform.

### 2.4. Sources of Artifacts and the Need for AI Enhancement

The physics that enable ultrasound imaging also give rise to artifacts. Key artifacts affecting sow pregnancy diagnosis include the following: (1) Reverberation caused by multiple reflections between strong, parallel reflectors (e.g., the probe-skin interface), creating false, repeating echoes deep to the real structure. (2) Acoustic shadowing, which is the attenuation of sound by a strongly reflecting or absorbing structure (e.g., bone or gas), creating a dark region behind it that can obscure underlying tissues. (3) Side lobes, which are off-axis energy from the transducer that creates false echoes from lateral targets, cluttering the image. These artifacts, as shown in the middle panel of Figure 1, can mimic or obscure early gestational sacs, leading to false negatives or positives. Traditional filters (e.g., bandpass filters for noise) are often ineffective as they cannot distinguish artifact patterns from true tissue textures based on spatial context. This limitation motivates the use of context-aware, adaptive AI models.

## 3. AI Algorithm for Biosensor Signal Enhancement

### 3.1. Dataset Construction and Enhancement for Robust Training

**Figure 3:**
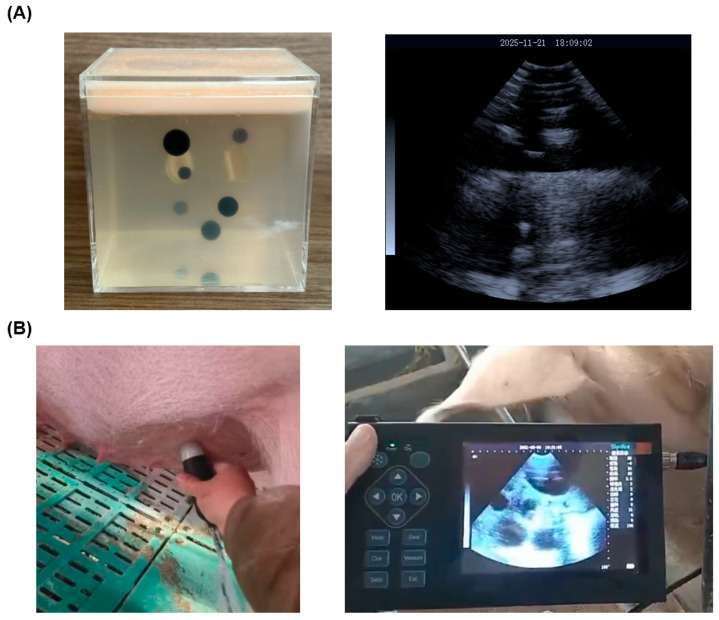
Data acquisition, artifacts, and AI enhancement results. (**A**) The ultrasound phantom model used for supplemental data collection, A raw B-mode image from the phantom. (**B**) photograph of acquisition of B-ultrasound image of a pregnant sow using an ultrasonic sector scanning probe, A raw B-mode image from a sow, showing a gestational sac (yellow circle) obscured by reverberation artifact.

A high-quality, diverse dataset is the cornerstone of an effective DNN. We constructed a dataset using the described ultrasonic biosensor from two primary sources: (1) Phantom Models: To supplement and control artifact presence, we used a visualized ultrasound-guided tumor puncture phantom model (Figure 3A), which provided consistent and complex artifact patterns. (2) In vivo Sow Scans: Ultrasound images were collected from sows at different pregnancy stages (1–12 weeks) in collaboration with a commercial pig farm (Figure 3B).

The acquired dataset comprised 500 images, which were divided into training (50%, 250 images), validation (30%, 150 images), test (20%, 100 images). The distribution across gestational weeks was as follows: weeks 1–4 (early gestation): 160 images; weeks 5–8 (mid gestation): 170 images; weeks 9–12 (late gestation): 170 images. This stratified division ensures balanced representation of different pregnancy stages across all sets. To ensure annotation consistency, three experienced veterinarians independently annotated a subset of 100 randomly selected images. Inter-annotator agreement was evaluated using Cohen’s Kappa coefficient, yielding an average Kappa of 0.86 (substantial agreement), confirming high annotation reliability. For quantitative evaluation, we created expert-corrected reference images by having three veterinary ultrasound specialists manually remove artifact regions using Photoshop’s content-aware fill tool, preserving only valid tissue structures. These expert-corrected references served as the ground truth for computing PSNR and SSIM metrics, providing a clinically meaningful benchmark for artifact suppression performance.

To prevent data leakage and ensure statistical independence, we implemented a three-level hierarchical splitting protocol: Animal-level separation: The 80 sows were first randomly divided at the individual animal level into training (40 animals), validation (24 animals), and test sets (16 animals). Session-level isolation: All ultrasound sessions from a given sow were kept entirely within one subset. Augmentation restriction: Data augmentation techniques were applied exclusively to training set images. No augmentation was performed on validation or test sets. This strict protocol ensures that no augmented variants of test images were used during training, and no information from test animals contaminated the training process. To combat overfitting and improve model generalization, we expanded the training dataset using the Image-Augmentation toolkit (https://github.com/Fafa-DL/Image-Augmentation, accessed on 20 November 2025). Techniques included random rotation (±10°), brightness/contrast adjustment (±15%), horizontal flipping, and addition of Gaussian noise. This process expanded our training dataset from 250 to 1000 annotated images, which were split into training, validation, and test datasets. The original 500 unique ultrasound frames were distributed across animals and sessions. After animal-level splitting, augmentation expanded the training set to approximately 1000 images while keeping validation (150 images) and test sets (100 images) completely independent and unaugmented. The images were meticulously annotated by team members with veterinary ultrasound expertise using LabelMe (5.2.0) software. Bounding boxes were drawn around key anatomical structures (e.g., gestational sac, uterus) and artifact regions.

### 3.2. YOLOv8 DNN Architecture for Intelligent Processing

**Figure 4:**
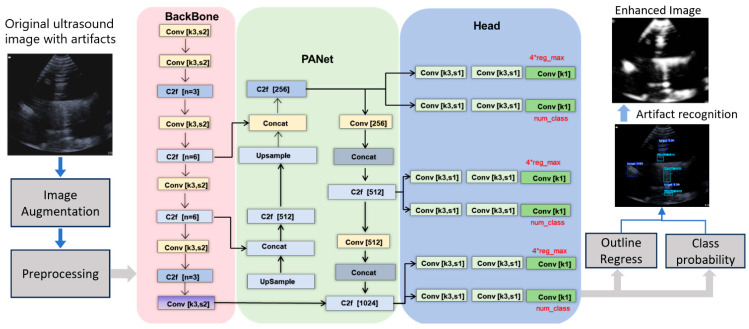
Architecture of the YOLOv8 DNN model for ultrasound enhancement. The diagram illustrates the three core components: the Backbone (C2f modules for feature extraction), the Neck (PAN-FPN for multi-scale feature fusion), and the Head (Decoupled head for separate classification and regression tasks). The input is an artifact-laden ultrasound image, and the output is a clean image with detected and localized structures (e.g., gestational sac).

We selected the YOLOv8 architecture for its excellent balance of speed and accuracy, making it suitable for real-time biosensing applications. The network structure, detailed in Figure 4, consists of three main parts:

(1) Backbone (Feature Extractor): Based on a modified CSPDarknet, it uses C2f modules (replacing the older C3 modules) to capture rich multi-scale features from the input image. The C2f module employs cross-stage partial connections for efficient gradient flow and feature reuse. A Spatial Pyramid Pooling Fast (SPPF) layer at the end aggregates multi-scale contextual information without significant speed loss.
(2) Neck (Feature Aggregator): Employs a Path Aggregation Network combined with a Feature Pyramid Network (PAN-FPN). This structure effectively combines high-resolution, low-level features (rich in spatial details like edges) with low-resolution, high-level features (rich in semantic meaning). This multi-scale fusion is crucial for detecting small, low-contrast targets like early gestational sacs.
(3) Head (Predictor): Uses a decoupled design, separating the tasks of classification (what is the object? “Gestational sac” (for example)) and regression (where is the object? bounding box coordinates). This separation leads to more accurate localization and classification compared to coupled heads.

The YOLOv8 architecture was adapted for simultaneous detection of artifacts and anatomical structures. Detected artifact regions were subsequently masked using a context-aware inpainting algorithm, while detected structures were annotated with bounding boxes. The final output is a composite visualization that suppresses artifacts while highlighting diagnostically relevant features.

The model was trained using a composite loss function combining Binary Cross-Entropy for classification and Complete Intersection over Union loss for bounding box regression. Training was performed on a system with an RTX 3060 GPU (NVIDIA, Santa Clara, CA, USA) using the PyTorch 2.6.0 framework, with a learning rate of 0.001 and a cosine annealing scheduler.

### 3.3. Error Analysis and Evaluation of Image Enhancement

**Figure 5:**
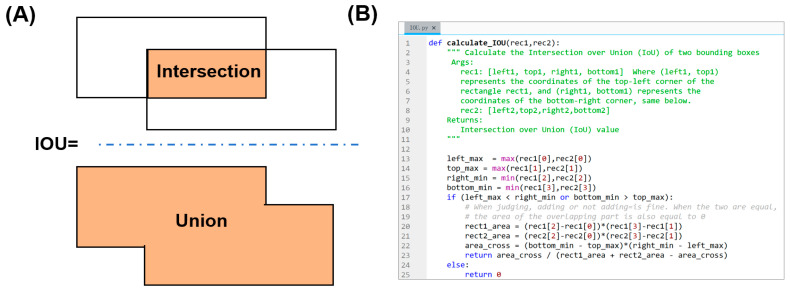
Error analysis using Intersection over Union (IoU). (**A**) A schematic diagram explaining the calculation of IoU. The dotted blue line represents division operation. (**B**) A screenshot of the IoU calculation script output, showing the computed IoU value for a sample prediction against its ground truth annotation. This quantitative metric was used to evaluate the precision of the AI model’s localization of gestational sacs.

A crucial part of our analysis was the calculation of localization error using Intersection over Union (IoU). IoU is defined as the area of overlap between the predicted bounding box and the ground truth box divided by the area of their union (Figure 5A). We implemented a Python (version: 3.9.7) function to calculate IoU by determining the coordinates of the intersecting rectangle and computing the respective areas (Figure 5B). An IoU of 1 signifies a perfect match, while 0 indicates no overlap.

## 4. Results and Discussion

### 4.1. Performance Comparison: AI vs. Traditional Methods

**Figure 6:**
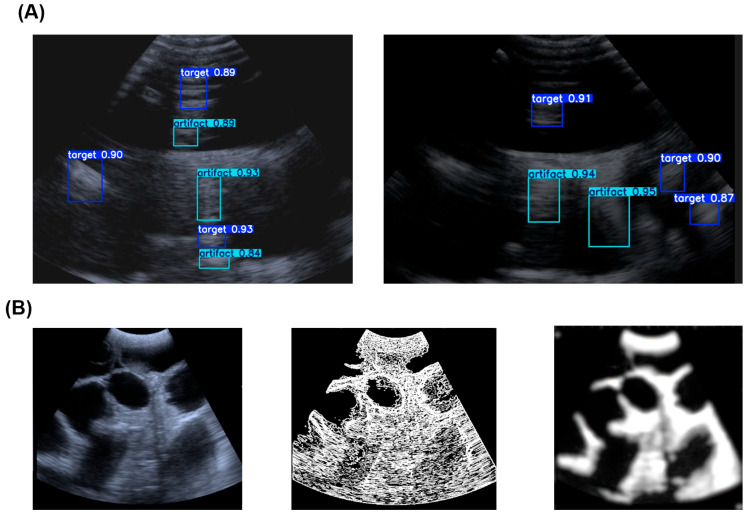
Comparison of enhancement methods. (**A**) Artifact recognition results using our AI model. (**B**) The first column shows the original, artifact-corrupted images. The second column shows results from traditional filtering methods, which struggle to remove artifacts without blurring critical details. The third column displays the outputs from our YOLOv8 model, demonstrating effective artifact removal and clear, sharp visualization of the gestational sacs.

The performance of our AI-enhanced biosensing system was quantitatively evaluated against traditional image filtering methods (e.g., wavelet denoising, median filtering). Figure 6 presents a qualitative comparison, clearly showing the AI model’s superior ability to suppress reverberation artifacts while preserving the structural details of the gestational sac. Traditional methods were implemented with carefully optimized parameters: Median filtering (5 × 5 kernel, applied iteratively 3 times); Wavelet denoising (Daubechies db4 wavelet, 4 decomposition levels, soft thresholding with universal threshold); Bandpass filtering (Butterworth 4th order, 3–7 MHz passband). For IoU computation with these non-detection methods, we first applied Otsu thresholding to generate binary masks, then extracted connected components as bounding boxes. These were compared against expert annotations using the standard IoU formula.

To ensure statistical independence in evaluation, all performance metrics were computed at the sow level by aggregating frame-level predictions within each scanning session and applying majority voting to determine the final diagnostic outcome per sow. Quantitative results, summarized in Table 1, confirm this qualitative assessment. Performance metrics reported in Table 1 represent animal-level accuracy, where a correct diagnosis was counted only if the majority of frames within a sow’s scanning session were correctly classified. This conservative evaluation approach prevents inflation of accuracy due to correlated frames. PSNR and SSIM were computed against expert-corrected reference images as described in Section 3.1. These metrics quantify how closely our enhanced visualizations approximate the artifact-free references created by clinical experts, providing objective measures of artifact suppression effectiveness. Our model achieved a mean IoU of 0.89, indicating highly accurate localization of the gestational sacs, compared to 0.65 for traditional methods. The PSNR of 34.2 dB and SSIM of 0.92 for the AI-enhanced images signify a much higher fidelity to the underlying tissue structure and a major improvement in image quality over the traditional approach (PSNR: 28.5 dB, SSIM: 0.72). Furthermore, as shown in Table 2, the model’s accuracy for detecting early pregnancy (1–4 weeks) was evaluated on a dedicated test subset of 160 early-gestation images, achieving 98.1% accuracy (157/160 correct), a critical improvement over the 76.4% accuracy (122/160 correct) of traditional methods, enabling more reliable early diagnosis.

Early pregnancy detection accuracy (1–4 weeks gestation) was defined clinically as: correct identification of at least one gestational sac with characteristic anechoic structure and double decidual sign, confirmed by subsequent farrowing outcome. Ground truth was established through consensus review by two veterinary experts and follow-up confirmation of pregnancy via farrowing records. A diagnosis was considered correct only if both the initial ultrasound interpretation and the eventual pregnancy outcome agreed.

### 4.2. Real-Time Processing Capability

For a biosensing system to be clinically viable, it must operate in real-time. We deployed the trained YOLOv8-n (nanosecond, the lightweight version) model on a standard laptop with CPU of Core i7-12700H (Intel, Santa Clara, CA, USA), and a GPU card of NVIDIA RTX 3060. The average processing time for a single 512 × 512-pixel ultrasound frame was 22 ms, which is well below the 30 ms threshold (corresponding to >33 fps) required for real-time, fluid video display. This demonstrates that the AI enhancement layer does not introduce disruptive latency and can be seamlessly integrated into the live biosensing workflow.

### 4.3. Training Dynamics and Model Confidence

**Figure 7:**
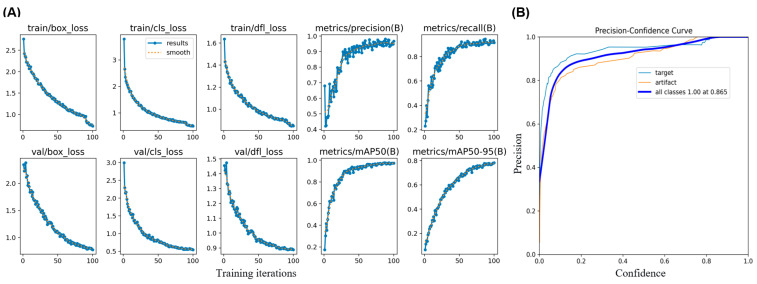
Model training performance and confidence analysis. (**A**) Training and validation loss curves (left *Y*-axis) and performance metrics (right *Y*-axis) over training iterations, showing stable convergence and improving accuracy. (**B**) Precision–Confidence curves for the model’s predictions. The curves for both ‘target’ (gestational sac) and ‘all classes’ show that precision increases with confidence, reaching 1.0 at a confidence threshold of ~0.87, indicating high model reliability for high-confidence predictions.

The training process was stable and efficient. Figure 7A shows the learning curves, where both training and validation losses for bounding box regression (box_loss), classification (cls_loss), and distribution focal loss (dfl_loss) decreased consistently and converged, indicating no signs of overfitting. Concurrently, the key detection performance metrics—precision, recall, and mean Average Precision at IoU = 0.5 (mAP50)—rose steadily and plateaued at a high level, confirming the model’s effective learning.

To assess the model’s confidence in its predictions, we analyzed the Precision–Confidence curve (Figure 6B). The curve shows that as the model’s prediction confidence increases, its precision (the fraction of correct identifications) also increases, reaching nearly 100% at high confidence levels. This relationship allows the system to flag low-confidence predictions for human review, enhancing overall diagnostic trustworthiness.

## 5. Conclusions

In this study, we have successfully developed and validated an intelligent ultrasonic biosensing system that synergistically combines a hardware biosensor (mechanical sector-scanning ultrasound) with a software AI intelligence (YOLOv8 DNN). This system directly addresses the long-standing challenge of acoustic artifacts in sow pregnancy imaging.

The key conclusion is that our AI-enhanced approach fundamentally outperforms conventional signal processing techniques. It delivers superior image quality, as evidenced by significant improvements in IoU, PSNR, and SSIM, and achieves a critical boost in early gestation detection accuracy. Furthermore, it accomplishes this without compromising the real-time performance essential for clinical and field use. By embedding physical understanding of ultrasound propagation into the data-driven learning process, we have created a model that intelligently discriminates between true tissue signals and artifact noise.

This work establishes a data-driven deep learning model that is not limited to veterinary ultrasonography. The framework of using a biosensor for data acquisition coupled with an acoustically informed DNN for intelligent signal enhancement is broadly applicable. While the proposed system demonstrates strong performance in artifact suppression and early pregnancy detection, it is important to acknowledge certain limitations. The current model was trained and tested on data collected from a single commercial breed (Landrace × Large White crossbred sows) under standardized feeding conditions. Variations in sow breed, body condition score, subcutaneous fat thickness, and dietary regimens may affect acoustic impedance and ultrasound image quality, potentially influencing model generalizability. Future work should include the following areas of investigation: (1) Multi-breed datasets should be built and controlled studies should be conducted to evaluate the robustness of the AI model across diverse swine populations and husbandry practices. Transfer learning and domain adaptation techniques could further enhance model adaptability. (2) Model interpretability should be enhanced using techniques like Grad-CAM to visualize decision processes. (3) The system should be extended to other domains such as human medical ultrasound (e.g., liver or kidney diagnostics) and industrial nondestructive testing. (4) Embedded versions should be developed for deployment on portable, low-cost ultrasound devices, thereby democratizing access to high-quality diagnostic imaging.

## Figures and Tables

**Table 1:** Quantitative performance comparison between traditional methods and the proposed AI model.

Metric	Traditional Methods	AI Model (YOLOv8)	Improvement
Mean IoU	0.65	0.89	+36.9%
PSNR (dB)	28.5	34.2	+20.0%
SSIM	0.72	0.92	+27.8%
Early Gestation (1–4 weeks) Accuracy	76.4%	98.1%	+21.7%
Processing Time (per frame)	>50 ms	22 ms	>56% faster

**Table 2:** Artifact-specific suppression performance of the AI model.

Artifact Type	IoU (Artifact Region)	PSNR Improvement (dB)	Visual Clarity Score (1–5)
Reverberation	0.91	+6.2	4.7
Acoustic Shadowing	0.87	+5.8	4.5
Side Lobes	0.89	+5.5	4.6

Visual Clarity Score: subjective evaluation by two veterinarians (5 = completely removed, 1 = no change).

## Data Availability

The data presented in this study are available on request from the corresponding author. The data are not publicly available due to privacy and ongoing research restrictions.
